# Case report of concurrent Fabry disease and amyotrophic lateral sclerosis supports a common pathway of pathogenesis

**DOI:** 10.3109/21678421.2016.1170150

**Published:** 2016-04-20

**Authors:** Alexander M. Beer, Johnathan Cooper-Knock, Sakina Fletcher, Sian Heledd Brown-Wright, T Pisharam Nandakumar, Pamela J. Shaw

**Affiliations:** ^a^ Sheffield Institute for Translational Neuroscience (SITraN), University of Sheffield; ^b^ Hull Royal Infirmary, Hull, UK

## Background

Fabry disease (FD) is a rare X-linked lipid storage disorder, resulting from deficiency of the enzyme alpha-galactosidase A (α-GAL). Consequent accumulation of globotriaosylceramide (GB3) in most cell types but particularly the vascular endothelium cells, produces progressive damage to the kidneys, skin, heart, and nervous system (1,2). Amyotrophic lateral sclerosis (ALS) is a neurodegenerative disorder characterized by progressive loss of motor neurons from the motor cortex, brainstem and spinal cord (3).

### Case history

**Figure 1.**
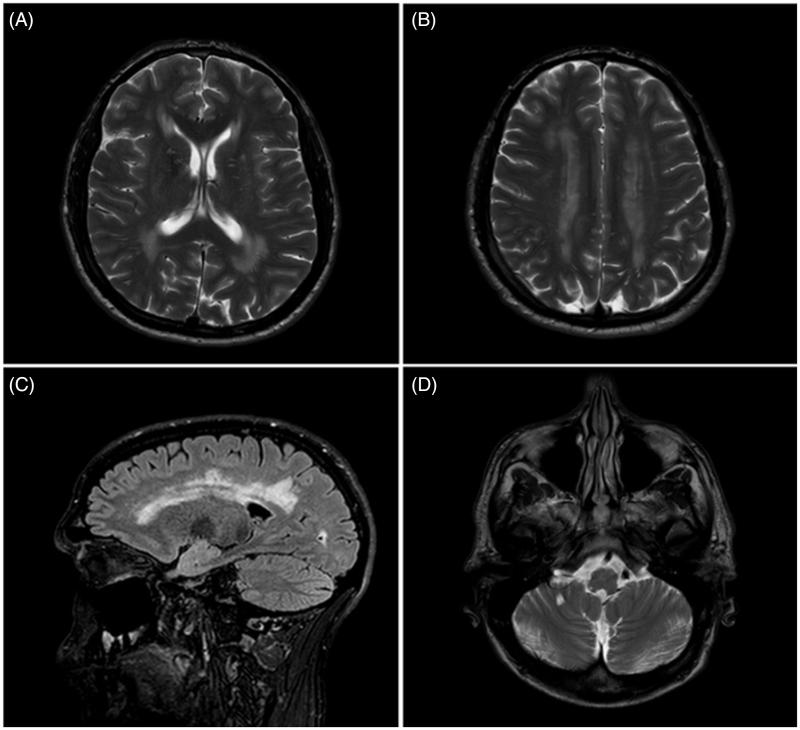
MRI brain scan from the patient with concurrent Fabry’s disease and amyotrophic lateral sclerosis. (A and B) T2 weighted transverse MRI scan showing symmetrical white matter change; (C) Sagittal FLAIR MRI scan showing white matter changes in the cerebral hemispheres; and (D) T2 weighted transverse MRI showing white matter changes in the brainstem.

We report a 38-year-old Caucasian male who presented with a five-year history of weakness and wasting in his right upper limb. He subsequently developed weakness in all limbs and the bulbar musculature. As a teenager, he experienced burning sensations in the distal parts of his limbs, and had significant diarrhoea. In his 20s he developed angiokeratomata in the groin area. Combined with cardiomyopathy and proteinuria, this led to a diagnosis of FD at the age of 35 years. Investigation revealed a c.299G > A mutation in the α*-GAL* gene and an abnormally low leukocyte activity of the α-GAL enzyme (1.96 pmol/punch/h). T1, T2 and FLAIR (fluid attenuated inversion recovery)-weighted MRI scans demonstrated widespread white matter changes (Figure 1). He has received Fabrizyme infusions every two weeks from the age of 37 years.

At the time of the initial presentation to our neuromuscular clinic, cognitive function and speech appeared normal. There was no wasting or weakness of the tongue, but occasional tongue fasciculations were noted. There was fasciculation and global muscle wasting of both upper limbs, more marked on the right, and worst in the hands. There was marked weakness of finger extension and of the ulnar and median-innervated intrinsic hand muscles bilaterally. The lower limb musculature was generally slim, but there was no observed focal wasting or fasciculation. Lower limb tone was increased. There was mild weakness of right hip flexion, but all other lower limb muscle groups were of normal power. The tendon reflexes were generally brisk and the left plantar response was extensor.

Serial electrophysiological studies demonstrated progressive changes with chronic and active denervation involving cervical, thoracic and lumbosacral regions that fulfilled the El Escorial electrodiagnostic criteria for ALS. Sensory nerve conduction was normal despite the burning sensation our patient had previously experienced; this is likely to be due to the reduced sensitivity of electrophysiology in recording from small myelinated or unmyelinated fibres. T1, T2 and FLAIR sequences demonstrated widespread white matter changes (Figure 1). Spinal MRI scans showed increased T2 signal in the left side of the cord at the level of C4. Genetic testing did not reveal a repeat expansion of *C9orf72*, a gene associated with ALS particularly in the presence of extramotor symptomology (4). Subsequently, seven years after he first noticed symptoms in the right upper limb, he has developed symptomatic respiratory muscle and bulbar weakness requiring both non-invasive ventilation and percutaneous gastrostomy.

## Discussion

**Figure 2.**
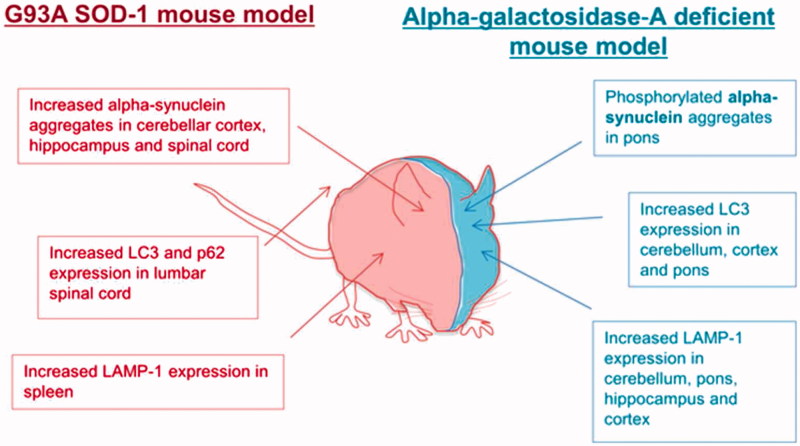
Autophagy is dysregulated in mouse models of both Fabry’s disease and SOD1-G93A amyotrophic lateral sclerosis.

FD is associated with neurological complications, but the motor system is often spared (5). To our knowledge, this is the first report of FD associated with an ALS-like phenotype. FD has been considered primarily a vasculopathy (6), which would not easily explain a motor neuron specific presentation. However, defects in autophagy have been observed in biosamples derived from patients with FD and in a number of neurodegenerative conditions including ALS. Study of renal tissue and fibroblasts from FD patients demonstrated increased staining of autophagy-associated markers (microtubule-associated protein 1A/1B-light chain 3 (LC3) and p62/SQSTM1) (7). Defects in autophagy have also been demonstrated in the brain of a mouse model of FD: increased immunoreactivity for LC3 and lysosomal-associated membrane protein 1 (LAMP-1) was accompanied by accumulation of protein aggregates and neurodegeneration (8). Several ALS-causing mutations have been identified in the autophagy pathway including OPTN and p62/SQSTM1, and impairment of autophagy has been linked to the hallmark pathology of ALS, the presence of cytoplasmic TDP-43 aggregates (9). The G93A-SOD1 transgenic mouse model of ALS shows histopathological similarities to the mouse model of FD, with increased staining of autophagy-associated markers (Figure 2) (10). We hypothesise that in our patient, impaired autophagy as a result of FD may have predisposed him to develop motor neuron injury and the clinical phenotype of ALS. There is some precedence in the association of Parkinson’s disease with another lipid storage disorder – Gauchers disease. If we are correct, it remains to be determined why an autophagy defect in FD leads specifically to ALS as opposed to another neurodegenerative disease phenotype, but this in itself might be an interesting source of putative therapeutic targets.

Alternatively, the motor neuron disorder in this patient may be the result of vascular insufficiency secondary to FD. However, the clinical picture is typical of ALS and the symmetry of the MRI white matter changes is not consistent with the marked asymmetry of the neuromuscular presentation.

This report highlights the findings in a single FD patient and as such could be a chance finding. However, given that both diseases are relatively rare – the incidence of both is 1–2 per 100,000 in the UK – a mechanistic link is worthy of consideration. Moreover, as FD treatment continues to improve life expectancy, it will be interesting to note whether other similar patients are reported in the future. This could have implications for the study and treatment of both FD and ALS.

## References

[CIT0001] Tuttolomondo A, Pecoraro R, Simonetta I, Miceli S, Pinto A, Licata G. (2013). Anderson-Fabry Disease: A Multiorgan Disease.. Current Pharmaceutical Design.

[CIT0002] MacDermot KD, Holmes A, Miners AH. (2001). Anderson-Fabry disease: clinical manifestations and impact of disease in a cohort of 98 hemizygous males.. Journal of Medical Genetics.

[CIT0003] Mitchell JD, Borasio GD. (2007). Amyotrophic lateral sclerosis.. Lancet.

[CIT0004] Cooper-Knock J, Hewitt C, Highley JR, Brockington A, Milano A, Man S (2012). Clinico-pathological features in amyotrophic lateral sclerosis with expansions in C9orf72.. Brain.

[CIT0005] Bersano A, Lanfranconi S, Valcarenghi C, Bresolin N, Micieli G, Baron P. (2012). Neurological features of Fabry disease: clinical, pathophysiological aspects and therapy.. Acta Neurologica Scandinavica.

[CIT0006] Tuttolomondo A, Pecoraro R, Simonetta I, Miceli S, Arnao V, Licata G (2013). Neurological Complications of Anderson-Fabry Disease.. Current Pharmaceutical Design.

[CIT0007] Chevrier M, Brakch N, Lesueur C, Genty D, Moll S, Djavaheri-Mergny M (2010). Autophagosome maturation is impaired in Fabry disease. Journal of Inherited Metabolic Disease.

[CIT0008] Nelson MP, Tse TE, O'Quinn DB, Percival SM, Jaimes EA, Warnock DG (2014). Autophagy-lysosome pathway associated neuropathology and axonal degeneration in the brains of alpha-galactosidase A-deficient mice.. Acta Neuropathologica Communications.

[CIT0009] Brady OA, Meng P, Zheng Y, Mao Y, Hu F. (2011). Regulation of TDP-43 aggregation by phosphorylation and p62/SQSTM1.. J Neurochem.

[CIT0010] Li L, Zhang XJ, Le WD. (2008). Altered macroautophagy in the spinal cord of S0D1 mutant mice.. Autophagy.

